# Stapes surgery in residency - the ufpr clinical hospital experience

**DOI:** 10.1016/S1808-8694(15)30125-7

**Published:** 2015-10-19

**Authors:** Adriano Ulisses Caldart, Igor Terruel, Dair Jocely Enge Júnior, Adriana Sayuri Kurogi, Maurício Buschle, Marcos Mocellin

**Affiliations:** a MD. 3rd year Otorhinolaryngology Resident, HC-UFPR; b MD. 3rd year Otorhinolaryngology Resident, HC-UFPR; c 4th Year Medical Student - HC-UFPR; d 4th Year Medical Student - HC-UFPR; e M.S. Assistant Professor of Otorhinolaryngology UFPR; f PhD. Full Professor of Otorhinolaryngology UFPR. Head of the Otorhinolaryngology Service - Paraná Federal University Medical School. Hospital das Clínicas da Universidade Federal do Paraná

**Keywords:** stapes surgery, otosclerosis, hearing loss

## Abstract

Surgery of the stapedius remains the established treatment for otosclerosis. Recent publications have showed that success in surgeries done by residents have decreased and hearing results are worse than those obtained by experienced otologic surgeons. **Aim:** To evaluate the experience of the otorhinolaryngology unit, Parana University, relative to stapes surgery done in the residency training program. **Material and method:** A retrospective study of 114 stapes surgeries done in the past 9 years in 96 patients. Audiometric results were analysed according to the Commitee on Hearing and Equilibrium guidelines and the Amsterdam Hearing Evaluation Plots. The improvement of the airway postoperative gap and thresholds were taken into account. **Results:** 96 patients were included, most of them female adults (67.7%) and white (93.7%). Stapedectomy was done in 50.9% of cases, mostly under local anesthesia and sedation (96.5%), using mostly the Teflon prothesis (37.7%). The surgical success rate was 50.88%, there was an 11.4% complication rate. **Conclusion:** Postoperative hearing gains considered as surgical success were inferior to published results in the literature, done by experienced surgeons.

## INTRODUCTION

Otosclerosis, also called otospongiosis, is a hereditary disease, more frequently found among women, in the rate of 2:1, in the age range between 20 and 40 years of age, and in Caucasians. It is very rare in African-descendants and very little frequent in the Asian population^1^, ^2^. It can be defined as an osteodystrophy located in the otic capsule of the temporal bone, causing stapes and vestibular ankylosis, and thus creating secondary effects on the hearing system (hearing loss and tinnitus) and vestibular symptoms (dizziness).

Modern otosclerosis surgery was developed by the pioneers M. Portmann e J. Shea, at the late 50’s, which they called, respectively, the “interposition” procedure and “oval window fenestration”^2^, ^3^. Right afterwards, the procedure was established and received the name “stapedectomy”, and it was adopted worldwide. Today, stapes surgery has become an established treatment for conductive hearing loss secondary to otosclerosis. It is a delicate microsurgery, however relatively simple when performed by experienced and trained surgeons. The procedure is usually very successful, bringing about an improvement in hearing.

In residency programs, the problem has been a reduction on the number of patients needing stapes surgery in recent years, creating a real difficulty in properly training resident physicians. This can be explained by the technological progress in the area of otology, especially in diagnosis and treatment, when we consider the number of otorhinolaryngologists trained to carry out this procedure, and by the current use of fluorine in the water^4^, ^5^. Recent publications have shown that the surgical success in stapes surgery carried out by resident physicians has dropped and the audiologic results have been worse than those obtained by experienced ear surgeons^5^, ^6^, ^7^, ^8^, ^15^.

This paper aims at assessing the experience of the otorhinolaryngology service in performing stapes surgery in the training program for resident physicians, defining the epidemiological profile of patients, the type of anesthesia, the technique employed, the type of prosthesis used, postoperative complications and audiologic results regarding the procedures performed by the residents.

## MATERIALS AND METHODS

The study was carried out after the retrospective analysis of the charts of four patients who underwent surgical treatment for otosclerosis, from January of 1996 to January of 2005. The procedures were carried out only as part of the medical residency training program in the aforementioned hospital, and residents in their last year of training have participated in these procedures, under direct supervision of the professor in charge.

Data obtained from the patient’s charts included: gender, age, race, surgical indication, the resident physician and the preceptor in charge, the type of anesthesia used, technique employed, type of prosthesis used, procedure time, postoperative complications and pre and postoperative audiologic results.

During this period, 151 stapes surgeries were carried out in 123 patients. There were three revisional surgeries, 9 surgeries in patients with concomitant systemic and otorhinolaryngological diseases, 25 patients without follow up and with incomplete data in their charts were taken off the study. Thus, we included 114 procedures in 96 patients.

Preoperative auditory assessment was carried out by means of the last tonal audiometry (in the frequencies of 0.25, 0,5, 1, 2, 3, 4, 6 and 8 kHz for air conduction; and 0,5, 1, 2, 3 and 4 kHz for bone conduction) and vocal audiometry held in the preoperative. Postoperative auditory evaluation was carried out through observing the results from the best audiometry carried out within a period of up to 6 months after surgery. Audiometric exams were performed with the CAT 741 audiometer by DICTON, in a soundproof booth by properly trained audiologists. When a bone or air conduction threshold was registered as immeasurable in a given frequency, they considered a value of 10 dB above the device’s capacity as the threshold.

We compared pre and postoperative audiometries in relation to the following points: bone and air conduction thresholds separately in each frequency; the average of air and bone conduction thresholds in the frequencies of 0.5, 1, 2 and 3 kHz, according to the guidelines of the Committee on Hearing and Equilibrium^12^; bone-air gap was obtained by subtracting the bone and air conduction threshold averages; the speech recognition threshold (SRT).

The surgical result was successfully classified in success and failure according to the guidelines of the Committee on Hearing and Equilibrium12. The result was classified as successful when the difference between postoperative air and bone conduction was below 10 dB or postoperative air conduction was below 30 dB. The remaining results were classified as failures. Individually, the surgical results were presented through the Amsterdam Hearing Evaluation Plots - AHEPs^13^.

Data statistical analysis was carried out by means of using the t-Student test for paired samples analysis. The data corresponding to the thresholds of the many frequencies analyzed suffered the square-root transformation. In order to compare both surgical techniques in relation to the presence of complications or in relation to the success of the surgical procedure, we used the Fisher’s Exact Test. P values <0.05 indicated statistical significance.

All the participants in the research freely signed the informed consent form and the University of Paraná Ethics in Research Committee previously approved this research protocol.

## RESULTS

Of the 96 patients included in the study, 31 (32.3%) were males and 65 (67.7%) were females, with ages varying between 14 and 69 years (mean value of 40.35 ± 11.20 years). In relation to race, 90 (93.7%) patients were Caucasians, 5 (5.2%) were blacks and only one (1.1%) patient was brown.

Of the 27 resident physicians belonging to the service, 23 participated in the stapes surgeries held during the time of the study, always under direct supervision of at least one preceptor. The number of surgeries per resident physician varied between 1 and 14, as it can be seen in Graph 1. The minimum number of operations carried out per year was of four (2003) and the maximum was 35 (1998).

**Chart 1:**
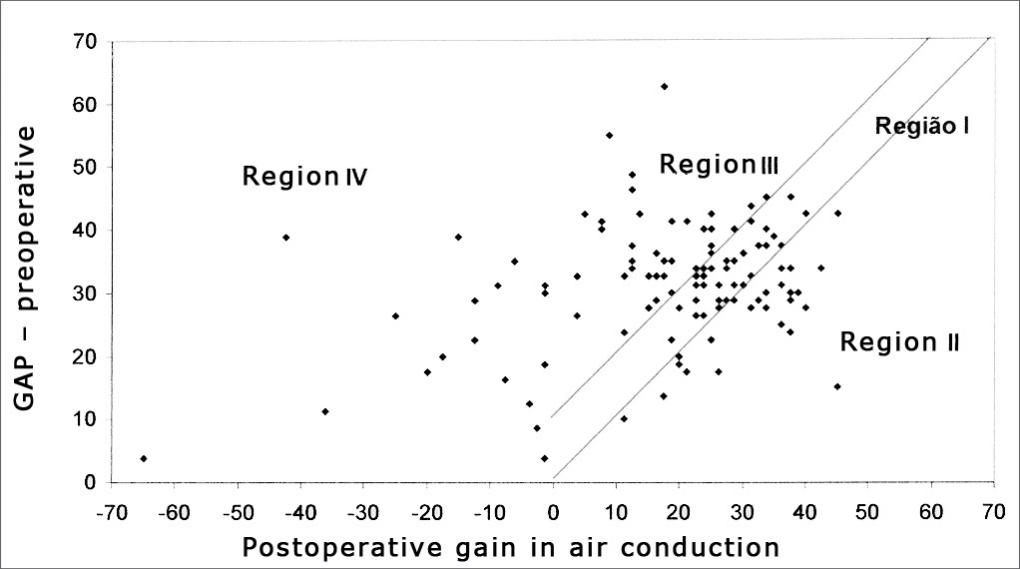
Number of surgeries per resident.

110 (96.5%) patients underwent the procedure under local anesthesia by a 2% lidocaine and 1:50000 adrenalin solution. These agents were followed by intravenous analgesia and sedation. General anesthesia was used in four (3.5%) patients who were also intubated and received intravenous and inhalator agents. Anti-vomiting drugs were administered at the end of almost all procedures.

As to the technique employed, of the 114 stapes surgeries carried out, 58 (50.9%) were stapedectomies and 56 (49.1%) were stapedotomies. Surgery success in relation to the technique employed can be seen on Table 1.

**Table 1:** Surgical success in relation to the technique used.

Surgery result	Technique	
	Stapedotomy	Stapedectomy
Failure (gap ^3^ 10 or air conduction PO ^3^ 30	28	28
	50.00%	48.28%
Success	28	30
	50.00%	51.72%

Total	56	58

The Teflon prosthesis were the ones most utilized in stapes surgeries (37.7%), followed by the gold prosthesis (29.8%) and the Schuknech prosthesis (22.8%). In the remaining, other types of prosthesis were used.

On Table 2, we present the mean pre and postoperative threshold values for air and bone conductions, of the air-bone gap and SRT.

**Table 2:** Pre and postoperative average thresholds of air and bone conduction, air-bone gap and SRT in the operated ears.

Frequency (kHz)	n	Preoperative	Postoperative	Difference	p^a^
Air conduction		Mean±SD	Mean±SD	Mean±SD	
0,5	114	61,27±12,83	39,04±19,43	22,24±20,37	<0,0001
1	114	57,41±13,04	35,75±18,51	21,67±20,63	<0,0001
2	114	52,28±14,99	36,23±18,28	16,05±19,43	<0,0001
3	114	51,27±15,91	38,20±19,06	13,07±19,76	<0,0001
4	114	52,11±17,01	43,46±20,59	8,64±19,72	<0,0001
Mean^b^	114	55,56±17,01	37,30±20,59	18,26±18,51	<0,0001
Bone conduction					
0,5	114	19,08±9,42	19,87±10,38	-0,79±8,69	0,5438
1	114	21,97±9,97	20,18±9,68	1,80±10,52	0,0565
2	114	26,27±11,25	24,52±11,62	1,75±12,52	0,0787
3	114	28,99±12,34	27,81±13,22	1,18±12,57	0,2457
4	114	30,09±13,84	31,10±14,12	-1,01±14,71	0,4790
Mean^b^	114	24,08±9,11	23,09±9,66	0,99±9,16	0,2523
Air-bone gapc^c^	114	31,48±9,79	14,21±13,17	17,27±15,26	<0,0001
Vocal audiometry					
SRT	107	57,77±12,36	38,45±16,80	19,32±19,91	<0,0001

a T-Student test for paired samples (p<0.05).

b Average of the frequencies 0.5, 1, 2 and 3 kHz.

c Difference between air and bone conduction mean values.

On Tables 3 and 4, we analyze the audiometric results according to guidelines from the Committee on Hearing and Equilibrium^12^.

**Table 3:** Audiologic results of stapes surgeries.

Surgical result	Frequency	Percentage
Failure (gap ^3^ 10 or PO air conduction ^3^ 30)	56	49,12
Success	58	50,88

Total	114	100,0

**Table 4:** Pre and postoperative air-bone gap.

gap	Preoperative Frequency (%)	Postoperative Frequency (%)
≤ 10	4 (3,51%)	55 (48,25%)
10,1 - 20	12 (10,53%)	28 (24,56%)
20,1 - 30	32 (28,07%)	16 (14,04%)
> 30	66 (57,89%)	15 (13,16%)

Total	114 (100,00%)	114 (100,00%)

In Graphs 2 and 3, the surgical results are presented according to the Amsterdam Hearing Evaluation Plots - AHEPs13. Graph 2 allows the assessment of the surgical procedure effect in bone conduction. In 92 cases (80.7%) there were no greater alterations than 10dB (region I) in postoperative bone conduction, in 16 cases (14.0%) there was an improvement in bone conduction that was greater than 10dB (region II), and in 6 ears (5.3%) there was a worsening in bone conduction that was greater than 10dB (region III). Graph 3 establishes the surgical success rate, correlating the gain obtained in air conduction with what was expected by the preoperative air-bone gap. In 35 (30.7%) operated ears we observed enough improvement in air conduction to make the postoperative air-bone gap lower than 10dB (region I), in 23 (20.2%) ears, there was gain in air conduction bigger than what was expected, meaning a concomitant improvement in bone conduction (region II), in 38 (33.3%) cases the change in air conduction was enough to make the postoperative air-bone gap lower than 10 dB (region III) and in 18 (15.8%) we noticed a worsening in air conduction after the surgery (region IV).

**Chart 2:**
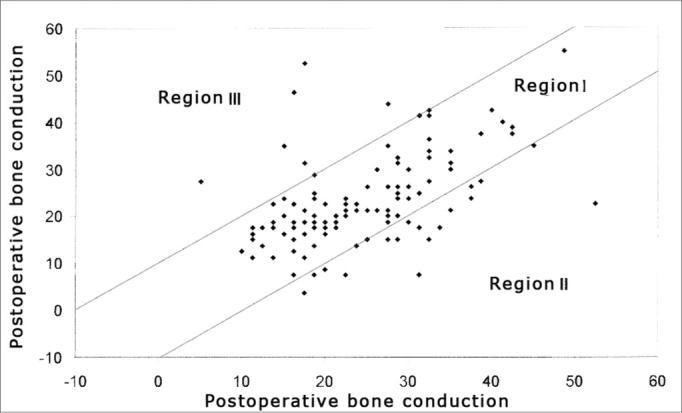
Graphic representation of pre and postoperative bone conduction threshold mean values according to AHEPs 13.

**Chart 3:**
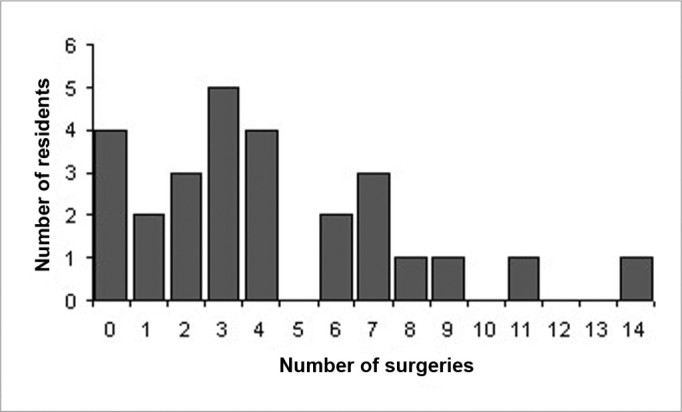
Graph representing the air conduction postoperative mean gain correlation with the preoperative air-bone gap. Results displayed in regions I and II represent surgical success.

As for postoperative complications, we had seven persistent tympanic membrane perforations, 5 persistent cases of vertigo and 3 cases of tinnitus, representing 11.4% of the total of surgeries performed. There were no cases of facial paralysis, perilymphatic fistulas, oval window granuloma or profound sensorineural hearing loss (“dead ear”).

## DISCUSSION

Otosclerosis is a disease that affects people in their adult age, reaching its peak incidence between 20 and 40 years. It is more frequent in women and in Caucasians^1^, ^2^. Burns and Lambert^15^ observed a higher incidence of cases of this disease in Caucasian females at a mean age of 46 years. Handley and Hicks^6^ noticed a higher incidence in Caucasian men at a mean age of 48.2 years. Most of the patients in this study were Caucasian females at a mean age of 40.35 years, which is different from what is described in most of the literature studied.

Stapes surgery is a delicate procedure that requires training and practice because of the many possibilities for complication and failure. Experienced and properly trained surgeons reach success rates of 90% or more^6^, ^15^. However, recent publications have shown that surgical success carried out in teaching institutions by resident physicians have reduced and the audiologic results have been worse when compared to those from experienced ear surgeons (Table 5)^5^, ^6^, ^7^, ^8^, ^15^. The possible cause for this would be a reduction in the number of patients who required stapes surgery, and thus, as it happens with any surgical procedure, the greater the number of surgeries performed, the greater the training and thus the success rate. Manu et. al.^19^ described that at least eight surgeries are necessary during the training phase, and seven more to reach a success rate of 75% and many more in order to have 90 to 95% of success in stapes surgery. In our University Hospital, of the 27 residents, only four perform more than the minimum number of surgeries recommended. Shapira et al.^8^ and Burns and Lambert^15^ described 2 essential conditions in order to achieve a good result in the surgeries performed by residents: experience with a large enough number of patients requiring middle ear surgery (including otosclerosis and Chronic Otitis Media) and a rigorous supervision from the professor in charge of the operating room. Handley et al.^6^ emphasized that an intensive training in the temporal bone dissection laboratory should be a pre-requisite for any middle ear procedure. Now, Mathews et al.^10^ observed that the progress in surgical techniques, especially the use of the small fenestra and the laser have increased the average success in stapes surgery performed by relatively inexperienced surgeons and thus they think it can be safely performed by resident physicians.

**Table 5:** Surgical results from stapes surgeries performed by surgeries.

Local	Author	Year	Surgeries/resident	Sucess
Virginia Health S. Center	Burns, Lambert 15	1996	-	89%
Kaiser Medical P. Center	Mathews, et. Al. 10	1999	71	87%
Kaplan Hospital - Israel	Shapira, et al. 8	1985	18	82%
Massachusetts Eye & Ear	Vernick 16	1986	6	78%
University of California	Engel, Schindler 9	1984	9	75%
Baylor College of Medicine	Backous 5	1993	2.9	68%
UAB	Handley, Hicks 6	1988	0.78	64%
Baylor College of Medicine	Coker, et al. 17	1988	2.7	64%
Miami	Chandler, Rodriguez-Torro 18	1983	6	62%

As to the surgical intervention, the type of anesthesia is an important factor and can depend on the surgeon’s preference. Most studies found in the literature 5, 6 show that there is no difference in relation to the type of anesthesia used in stapes surgery; however, they advocate the use of general anesthesia in medical residency programs, because it facilitates the performance of this surgery by physicians in training, facilitating the communication with the professor and reducing concerns regarding surgery duration. Burns and Lambert^15^ described that local anesthesia and sedation is more advantageous because it reduces the theoretical risk of prosthesis shifting during the time when the patient has the orotracheal tube removed, reduces the episodes of nausea and vomit in the postoperative period and allows the patient to tell the physician about his hearing at the end of the procedure, thus providing the surgeon with facts to discuss surgical results. In our study, almost all the surgical procedures were carried out under local anesthesia and sedation and this establishes our alignment with the approach of these authors.

Another important factor is related to the technique employed, especially when complications happen. Backous et al.^5^, Burns and Lambert^15^ and Lesinski^22^ described in their papers a stapedectomy in most of these cases, stressing the fact that it can maximize the performance of resident physicians and, consequently, increase the success rate associated with the procedure. Mathews et al.^10^ and De Bruijn et al.^14^ reported that they performed stapedotomies in almost all their cases, showing a very low likelihood of prosthesis migration, which corresponds to the most common cause for failure. In the surgeries performed in our institution during the study period, stapedectomy prevailed over stapedotomy; however, the latter has been performed more frequently in the last 4 years.

We did not notice statistically significant differences in success rates when we considered the surgical technique employed. In studies by Warmerdam et al.^11^ and Sedwick et al.^21^ they also did not observe statistically significant differences in surgical success rates when they considered the techniques employed.

There are many types of prosthesis in the market; however, the ideal one is yet to be discovered. The best prostheses are the ones which are biocompatible, of easy handling, and that properly fits the incus in order to transmit the vibrations to the oval window^20^. In this study, the Teflon prosthesis was the one most utilized. This large prosthesis variability makes it difficult to compare studies that describe the routine of other institutions.

In the current literature about stapes surgery, there is a great variation in audiologic parameters and criteria utilized to establish surgery success rate, and this makes it difficult to compare the results presented by different authors. Moreover, vocal audiometry results are rarely taken into account, which is not in agreement with the major goal of this procedure, which is to improve patient communication, in other words, speech recognition threshold (SRT). In the present investigation, we tried to minimize these differences by using the guidelines from the Committee on Hearing and Equilibrium^12^, which propose standardization in middle ear surgeries success rate evaluations, thus facilitating the comparison between studies. When we assessed surgical success that corresponds to a postoperative air-bone gap below 30 dB, we noticed a success rate of 50.88%. In 72.81% of the cases, we observed a postoperative air-bone gap bellow 20 dB, a successful procedure indicator in some literature studies. This success rate is comparatively below the ones found in the literature, as we can see on Table 5. This can be explained by the resident surgeons’ inexperience because of the reduced number of these procedures carried out during their training. There was an improvement in speech recognition threshold (SRT), which is in agreement with many studies revised in the literature^13^, ^15^.

In order to present the middle ear surgical results in large samples, in a simple and clarifying way, De Bruijn et al.^13^ developed a data analysis method called Amsterdam Hearing Evaluation Plots - AHEPs. This method brings about a visual representation of the auditory results for each ear individually after the middle ear procedure. The major advantage of his procedure is that favorable or unfavorable results in terms of the surgical success can be easily identified and the ears that suffered cochlear damage from surgery can be recognized. A worsening in postoperative bone conduction represents cochlear damage. On the other hand, there may be bone conduction improvement after the procedure because of the so-called Carhart effect. This means an improvement in the inertial component (skull vibration with a rigid body) of bone conduction sound transmission in the postoperative and postoperative bone conduction thresholds better corresponding to the true functional status of the inner ear1. In this study, cochlear damage happened in only six cases (5.3%), and such rate that is above the one described by De Bruijn et al.^13^, however lower than the one observed by Frias et al.^22^.

Thus, as success rates fall, postoperative complications amount^15^. We had complications in 11.4% of all the surgeries performed, however with not one case of facial paralysis, perilymphatic fistula or profound sensorineural hearing loss. These data are similar to the ones found in many studies published in the literature^18^, ^10^, ^5^.

Of course that during medical residency, all residents must have the opportunity to perform stapes surgery. However, those who do not intend to be ear surgeons later on must be encouraged to refer their cases to the ones who intend to be ear surgeons. Thus, having a patient who requires surgery, the resident must educate the patient in regards of he success rates attained in their residency program and not expect results attained by preceptors or experienced surgeons. Such behavior must be encouraged in services, which receive few stapes surgery cases.

## CONCLUSION

Most patients submitted to stapes surgeries in our otorhinolaryngology service were adults (mean value: 40.35 ± 11.20), females (67.7%) and Caucasians (93.7%).

The surgical technique that was most employed in our service during the time of the study was stapedectomy (50.9%), and almost all the patients were operated under local anesthesia and sedation (96.5%). In these surgeries, the Teflon prosthesis was the one most frequently used (37.7%). There was no statistically significant difference in success rate as far as the technique is concerned.

Our postoperative surgical success rate (50.88%) was lower than those reported in the literature by experienced surgeons, and there were no statistically significant differences when the complication rates were compared (11.4%).
